# Facing the challenge of viral mutations in the age of pandemic: Developing highly potent, broad‐spectrum, and safe COVID‐19 vaccines and therapeutics

**DOI:** 10.1002/ctm2.284

**Published:** 2021-01-12

**Authors:** Shan Su, Qian Wang, Shibo Jiang

**Affiliations:** ^1^ Key Laboratory of Medical Molecular Virology (MOE/MOH/CAM), School of Basic Medical Sciences Shanghai Institute of Infectious Disease and Biosecurity Fudan University Shanghai China

**Abbreviations**
ACE2: angiotensin‐converting enzyme 2
cGAMP: 2′,3′‐cyclic guanosine monophosphate‐adenosine monophosphate
CoG‐UK: COVID‐19 Genomics Consortium UK
COVID‐19: coronavirus disease 2019
HCoV: human coronavirus
MERS‐CoV: Middle East respiratory syndrome coronavirus
NTD: N‐terminal domain
ORF: open reading frame
PS: pulmonary surfactant
RBD: receptor binding domain
RdRp: RNA‐dependent RNA polymerase
SARS‐CoV‐2: severe acute respiratory syndrome coronavirus 2
SARSr‐CoV: SARS‐related coronavirus
UK: United Kingdom

Severe acute respiratory syndrome coronavirus 2 (SARS‐CoV‐2) has ravaged the globe throughout 2020. Very recently, the UK imposed further lockdown restrictions upon the discovery of a new SARS‐CoV‐2 lineage, B.1.1.7, which appears to have stronger human‐to‐human transmissibility. The COVID‐19 Genomics Consortium UK (CoG‐UK) found that the number of B.1.1.7‐infected cases has grown markedly since September, 2020, and that this lineage can account for an increased proportion of clinical cases in several regions of England.^1^ In a press conference, the Chief Science Adviser of CoG‐UK remarked that “the slew of mutations may have increased the virus’ transmissibility by 70%."^2^ This news immediately provoked widespread concern and intensive discussion.

The B.1.1.7 lineage contains an unusually large number of genetic changes, more than previous SARS‐CoV‐2 isolates, including 14 non‐synonymous mutations and three deletions in ORF1ab, spike protein, ORF8, and nucleocapsid protein. Among them, N501Y in the receptor binding domain (RBD) of the spike (S) protein is reported to be associated with coronavirus adaption to wild‐type mice and increased binding affinity to murine angiotensin‐converting enzyme 2 (ACE2), the receptor of SARS‐CoV‐2.^3^ Moreover, a deep mutational scanning of SARS‐CoV‐2 RBD revealed that N501Y, as well as N501F and N501T, could enhance the affinity of RBD to human ACE2,^4^ indicating that lineage B.1.1.7 may have increased ability to infect host cells. Although not identical to B.1.1.7, clinical variants containing N501Y have also been identified in several other countries, including South Africa and the Netherlands.^2^ The origin of the N501Y mutation remains elusive. In two independent studies, SARS‐CoV‐2 variants carrying N501Y were isolated from serial passaging in aged Bagg Albino/c mice and a persistent infection of SARS‐CoV‐2 in an immunocompromised host, respectively,^3^, ^5^ indicating that B.1.1.7 may come from the evolution of SARS‐CoV‐2 in an immunocompromised host.

Another noteworthy mutation in B.1.1.7 is the H69 and V70 deletion (69‐70del) in the N‐terminal domain of the spike protein. A variant containing 69‐70del and D796H mutations has been found to escape from neutralizing antibodies (NAbs) in an immune suppressed patient treated with convalescent plasma.^6^ Therefore, the 69‐70del mutation may further endow lineage B.1.1.7 with the ability to resist antibody therapy. In November, the rapid and uncontrolled spread of SARS‐CoV‐2 variants carrying 69‐70del in mink and then human has led to a cull of millions of minks in Denmark.^7^

Except for the N501Y and 69‐70del mutations, P681H, one of four residues comprising the furin cleavage site in the spike of SARS‐CoV‐2, may affect viral entry.^8^ The ORF8 Q27stop mutation, an analog of the mutant prevalent in Singapore during the early pandemic, may have changed viral pathogenicity.^9^ At this writing, the effects of the B.1.1.7 lineage, in combination with the above‐noted mutations, on the COVID‐19 pandemic remain elusive. This calls for a reprioritization scheme that includes the following issues. First, we must ask if lineage B.1.1.7 is, indeed, more infectious, as well as more lethal, than wild‐type SARS‐CoV‐2, and whether the rapid spread of B.1.1.7 is a function of increased infectivity and pathogenicity. Second, it has been shown that a recombinant RBD vaccine can still protect mice against infection by a SARS‐CoV‐2 variant with N501Y mutation.^3^ However, we do not know if the currently developed COVID‐19 vaccines registered for distributions in different countries or authorized for emergency use, e.g., BNT162 mRNA vaccine from Pfizer‐BioNTech and mRNA‐1273 vaccine from Moderna, as well as antibodies (e.g., Regeneron's REGN‐COV2), will have efficacy against B.1.1.7. Third, the faster spread of B.1.1.7 in the UK was a conclusion reached on the basis of an analysis of 126 219 genomes from positive samples generated by the CoG‐UK, which has closely monitored genetic changes in the SARS‐CoV‐2 genome since April 2020.^10^ By logical extension, other countries should consider establishing such surveillance and tracking system able to quickly detect mutations in current SARS‐CoV‐2, as well as emerging strains of other coronaviruses.

SARS‐CoV‐2, as an RNA virus, is constantly mutating, and the large‐scale administration of COVID‐19 vaccines may exert selective pressure on the virus to further evolve into a vaccine‐resistant strain. In addition, the SARS‐related coronaviruses (SARSr‐CoVs) from bats may cause outbreaks of novel SARS‐like diseases in the future. Therefore, in previous writings, we have called for the development of effective, broad‐spectrum, and safe anti‐coronavirus vaccines and therapeutics.^11^, ^12^ The emergence of B.1.1.7 further emphasizes the wisdom of this step, and its methodology can be summed up as follows.

Generally, therapeutics and vaccines targeting conserved epitopes are likely to be broadly effective to diverse mutant strains and subtypes. For example, our group identified a peptide, termed as EK1. This peptide targets the conserved HR1 domain of human coronavirus, and it has shown efficacy in inhibiting infection of all circulating human coronaviruses tested, including SARS‐CoV, MERS‐CoV, HCoV‐229E, HCoV‐NL63, and HCoV‐OC43, as well as three bat SARSr‐CoVs in vitro. Intranasal administration of EK1, or its analog, EK1C4, exhibited prophylactic and therapeutic effects against HCoV‐OC43 and MERS‐CoV infection in susceptible mouse models.^13^ Notably, this broad‐spectrum coronavirus inhibitor was developed before the COVID‐19 outbreak and also showed efficacy against SARS‐CoV‐2, suggesting that EK1 and EK1C4 could be used to prevent and treat infection by the currently circulating and future emerging coronaviruses.^14^, ^15^ We believe that another conserved antiviral target, such as the coronavirus main protease (M^pro^) or RNA‐dependent RNA polymerase nsp12, can also be a promising target for the development of effective pan‐coronavirus therapeutics or prophylactics.

Such conserved target proteins can be used to design broad‐spectrum therapeutics, but they are less satisfactory for developing NAbs and vaccines because they lack exposure on the free virions. The spike protein of SARS‐CoV‐2 covers the viral surface and is, therefore, good bait to fish out antibodies and antigens for vaccine design. However, the spike protein, especially the RBD of SARS‐CoV‐2 is under high selective pressure and mutates frequently.^1^ Therefore, new strategies for broad‐spectrum vaccine design are needed. It was reported that antibodies isolated from SARS patients can cross‐react with SARS‐CoV, SARS‐CoV‐2, and SARSr‐CoVs by binding to the RBD,^16^ indicating that some conserved epitopes may exist in RBD. Tai et al reported that the lipid nanoparticle‐encapsulated RBD‐based mRNA COVID‐19 vaccine and RBD‐Fc‐based subunit vaccine could elicit potent NAbs able to inhibit the infection of live and pseudotyped SARS‐CoV‐2 and SARS‐CoV pseudoviruses expressing S proteins of human strains Tor2 and GD03, as well as palm civet strain SZ3.^17^ Our recent study demonstrated that the recombinant RBD‐based subunit vaccine elicited potent NAbs that inhibit the infection of live and pseudotyped SARS‐CoV‐2, and SARS‐CoV‐2 pseudovirus with natural mutations in RBD, as well as SARS‐CoV and bat‐SARSr‐CoV‐WIV1 pseudoviruses.^18^ These results further confirmed the existence of conserved and neutralizing epitopes within the RBD. Optimization of RBD by masking the non‐NAb and non‐conserved epitopes with glycosylation is expected to further improve its protective immunity and breadth.^19^

Some adjuvants can also help improve the protective breadth of vaccines. In collaboration with Wu and colleagues at Harvard Medical School, we identified pulmonary surfactant (PS)‐biomimetic liposomes encapsulating 2′,3′‐cyclic guanosine monophosphate‐adenosine monophosphate (cGAMP), an agonist of the interferon gene inducer STING (stimulator of interferon genes), as a novel adjuvant. PS‐GAMP‐adjuvanted H1N1 vaccine elicited strong cross‐protection against distant H1N1, H3N2, H5N1, and H7N9 strains for at least 6 months.^20^ Interferons are the chief components of innate immunity against broad viral infections. Therefore, other adjuvants originated from the innate immune pathways may also be able to induce broad protection against divergent human coronaviruses.

Currently, Regeneron's REGN‐COV2 and Lilly's LY‐CoV555, both of which are cocktail therapies comprising RBD‐reactive antibodies, have been granted Emergency Use Authorization (EUA) for COVID‐19 by the US FDA. As noted above, COVID‐19 mRNA‐based vaccines from Pfizer/BioNTech and Moderna are currently in production. It is expected that the sensitivity of B.1.1.7, as well as that of emerging mutant strains, to these vaccines and antibodies will be continuously monitored. Meanwhile, as also suggested above, public confidence in the stability of the public health system will be tested as countries move toward establishing a surveillance and tracking system, alongside developing broad‐spectrum therapeutics and vaccines, for currently circulating and future emerging coronaviruses.

## FUNDING INFORMATION

This study was supported by grants from the National Natural Science Foundation of China (grant numbers: 82041025 and 81701998) to Shibo Jiang and Qian Wang, respectively.

## CONFLICT OF INTEREST

The authors declare that there is no conflict of interest that could be perceived as prejudicing the impartiality of the research reported.

## AUTHOR CONTRIBUTIONS

Shan Su drafted the manuscript. Shibo Jiang and Qian Wang conceived the study idea and modified the draft.

## AVAILABILITY OF DATA AND MATERIALS

All data generated or analyzed during this study are included in this published article.
